# Proteomics Reveal the Effect of Exogenous Electrons on Electroactive *Escherichia coli*

**DOI:** 10.3389/fmicb.2022.815366

**Published:** 2022-04-06

**Authors:** Jiao Feng, Jia Feng, Chunqiu Li, Sheng Xu, Xin Wang, Kequan Chen

**Affiliations:** State Key Laboratory of Materials-Oriented Chemical Engineering, College of Biotechnology and Pharmaceutical Engineering, Nanjing Tech University, Nanjing, China

**Keywords:** *Escherichia coli*, exogenous electrons, proteomics, differentially expressed proteins, bioelectrochemical system

## Abstract

Microbial cells utilizing electricity to produce high-value fuels and chemicals are the foundation of the biocathodic bioelectrochemical system. However, molecular mechanisms of electron transfer and utilization have not been elucidated. In this work, *Escherichia coli* engineered by introducing the Mtr pathway from *Shewanella oneidensis* exhibited stronger electrochemical activity than control and could utilize exogenous electrons to stimulate metabolite profiles and boost succinate production in the bioelectrochemical system. Proteomic analysis and real-time PCR were performed to investigate the effect of exogenous electrons on electroactive *E. coli.* Bioinformatics analysis suggested that the proteins of molecular function associated with oxidoreductase activity, 4 iron, 4 sulfur([4Fe-4S]) cluster binding, iron-sulfur cluster binding, and metal cluster binding were positively affected by exogenous electrons. Moreover, mapping to the Kyoto Encyclopedia of Genes and Genomes pathway database showed that the up-regulated proteins were mainly involved in metabolic pathways of tricarboxylic acid cycle, pyruvate metabolism, and nitrogen metabolism pathway, providing support for the metabolic balance of microbial cells shifting toward reduced end-products due to electron utilization. Using a biochemical method, the *ompF*-overexpressed strain was employed to investigate the function of the channel protein. These findings provided a theoretical basis for further improving electron transfer and utilization efficiency, and contributed to the potential applications of the bioelectrochemical system.

## Graphical Abstract

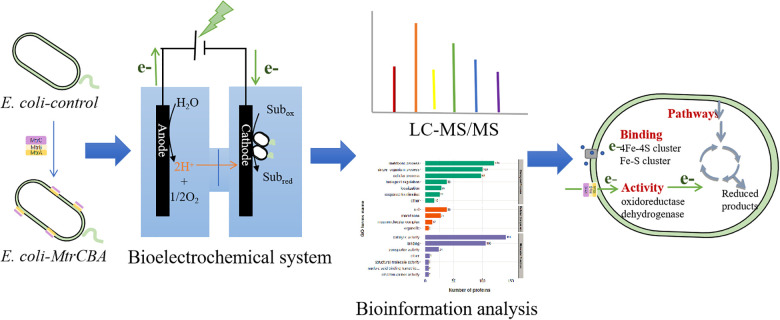

## Introduction

In a bioelectrochemical system (BES), microorganisms exchange electrons directly or indirectly with solid electrodes via extracellular electron transfer to manufacture or utilize renewable resources (Kracke et al., 2015; Sun et al., 2020). Biocathodic BESs, e.g., those using microbial electrosynthesis (MES) or cathodic electro-fermentation (CEF), are considered to be a green, sustainable, and attractive novel technology. In such BESs, high-value biofuels and chemicals are converted by microbial catalysis from CO_2_ or other substrates with electricity as the energy source, thereby achieving renewable energy storage in chemical bonds (Kracke et al., 2015; Patil et al., 2015;

Jiang and Zeng, 2018; Kracke et al., 2018). One advantage of these BESs is that low-cost carbon sources or wastes can be utilized by microorganisms as substrates. Another main advantage is the ability of microorganisms to utilize clean electricity to boost biofuel and chemical generation.

Microorganisms are the catalytic center of biocathodic BESs, whose parameters, activities, and functions are related to chemical production, reactor performance, and efficiency (Kracke et al., 2015; Kokko et al., 2016; Kracke et al., 2018). Electrons are transferred from cathodes into microbial cells, and then participate as essential elements in intracellular redox reactions and microbial metabolism (Sun et al., 2020). This process is applied to break through intracellular redox or energy limitations and shift metabolite profiles toward increasing terminal products of interest (Kracke et al., 2015; Kracke et al., 2018; Wu et al., 2019). Therefore, the ideal biocathodic BES must require microbial cells to take up electrons and efficiently incorporate them into a target cellular metabolic pathway (Kracke et al., 2018). Increasing efforts have focused on creating or promoting electron transfer (Tefft and TerAvest, 2019; Wu et al., 2019; Zhao et al., 2020). However, the molecular mechanism of electron transfer and effects on microbial cells are not clear (Korth and Harnisch, 2019; Sun et al., 2020). Existing research data, especially of biocathodes, are insufficient to conduct a comprehensive analysis, hindering further engineering of microorganisms to use electrons for efficiently generating target products, thereby limiting practical applications of biocathodic BES.

Proteomics technology is the most common method for conducting large-scale analysis on proteomic profiles and screening different target proteins (Zhang et al., 2020). A complete map of expression states derived from quantitative protein information provides insights into biological processes and helps reveal biological mechanisms (Schmidt et al., 2016; Zhang et al., 2020). The proteomes of many typical model microbes have been supported by numerous resources and databases. The *Escherichia coli* proteome is by far the most well defined bacterial proteome and is thus extensively used for studying general features of the prokaryotic proteome (Soufi et al., 2015; Fortuin et al., 2020).

Exploiting *E. coli* to produce electrosynthetic chemicals by engineering the electron transfer pathway is an important research area in BESs, which could make up for some deficiencies of electrochemically active bacteria and broaden the application prospects of biocathodic BES (Kracke et al., 2015; Mayr et al., 2019; Wu et al., 2019). The Mtr pathway from *Shewanella oneidensis* MR-1 is one of the most extensively studied electron transfer pathways and has been successfully constructed in *E. coli* (Jensen et al., 2010; Ross et al., 2011; Wu et al., 2019). The major components of this pathway are MtrCAB complexes (encoded by the *mtrCAB* cluster) containing MtrC, MtrB, and MtrA proteins (Jensen et al., 2010). We previously introduced the Mtr pathway into *E. coli* cells by expressing *mtrCAB* from *S. oneidensis* and the engineered *E. coli* could use the Mtr pathway to transfer electrons from the cathode into *E. coli* cells (Feng et al., 2020a). However, the molecular mechanism of electron utilization on *E. coli* cells has not been elucidated.

Based on these previous findings, this work first compared the metabolite profiles and electrochemical activity of engineered *E. coli* and the control strain without the Mtr pathway in the BES driven by electricity. The engineered *E. coli* exhibited better electrochemical activity and could use exogenous electrons to stimulate metabolite profiles, increasing succinate production by 18%. Subsequently, real-time PCR (RT-PCR) and proteomics methods were used to comprehensively analyze the specific gene expression changes and the differentially expressed proteins (DEPs) of *E. coli*. Moreover, the channel protein OmpF was found to be up-regulated in the proteomic data of subcellular location, and thus identified by further gene expression, showing the increased redox reactions between the electrode and the *E. coli* cells.

## Materials and Methods

### Bacterial Strains and Culture Conditions

Bacterial strains and plasmids are listed in Table 1, and primers are in Supplementary Material. The recombinant strains used in this work were formed by transforming the corresponding recombinant plasmids (Table 1). The recombinant plasmid pCWJ-*ompF* was constructed by ligating *ompF* gene fragment into pCWJ between the *Eco*RI and *Sac*I sites. The *ompF* gene fragment was amplified by PCR using ompF-1 and ompF-2 as primers and the *E. coli* BA102 genome as the template.

**TABLE 1:** Strains and plasmids.

Strains/plasmids	Relevant characteristics	References/source
*E. coli* Trans1-T1	Used for cloning	TransGen Biotech, Beijing, China
*E. coli* BA102	Used as host strain	Feng et al., 2020a
*E. coli-control*	*E. coli* BA102 harboring plasmid pCWJ-*ccmA-H*	Feng et al., 2020a
*E. coli-MtrCBA*	*E. coli* BA102 harboring plasmids pBBR1MCS-5-*mtrCAB* and pCWJ-*ccmA-H*	Feng et al., 2020a
*E. coli-mtr-ompF*	*E. coli* BA102 harboring plasmids pBBR1MCS-5-*mtrCAB* and pCWJ-*ompF*	This work
*E. coli-mtr*	*E. coli* BA102 harboring plasmids pBBR1MCS-5-*mtrCAB*	This work
pBBR1MCS-5-*mtrCAB*	pBBR1MCS-5 carrying the *mtrCAB* gene of *S. oneidensis* MR-1 under constitutive *plac* control; Gm*^r^*	Feng et al., 2020a
pCWJ-*ccmA-H*	pCWJ carrying the *ccmABCDEFGH* gene of *E. coli* K12 under constitutive *ptrc* control; Cm*^r^*	Feng et al., 2020a
pCWJ*-ompF*	pCWJ carrying the *ompF* gene of *E. coli* BA102 under constitutive *ptrc* control; Cm*^r^*	This work

A suspension of overnight-activated strains was inoculated into 100 ml of 2 × Yeast extract and Typeptone (2 × YT) medium in 500 ml flasks supplemented with the corresponding antibiotics and isopropyl-beta-D-thiogalactopyranoside (0.1 mM chloramphenicol for *E. coli-control*; 0.1 mM gentamicin for *E. coli-mtr*; and 0.1 mM chloramphenicol and 0.1 mM gentamicin for *E. coli-MtrCBA* and *E. coli-mtr-ompF*). The cultures were grown at 30°C with shaking at 200 rpm for 12–14 h. The suspended cells harvested by centrifugation were saved for further studies.

### Construction of Bioelectrochemical Reactors and Electrochemical Analysis

The harvested cells dispersed into the cathode chamber (OD_600_ = 1) of the dual chamber “H” bioelectrochemical reactors with an internal volume of 100 mL and fine carbon felts as the cathode and anode electrodes. The anode electrolyte and cathode medium were described in our previous work (Feng et al., 2020a). The anodic electrolyte contained 25 mM NaCl, 15.7 mM NaH_2_PO_4_⋅2H_2_O, 7.0 mM NaHPO_4_⋅12H_2_O, and 0.02% dithiothreitol (DTT) (filtered through a 0.22 nm syringe filter before use). The cathodic medium was composed of 47.8 mM NaH_2_PO_4_⋅2H_2_O, 88 mM NaHPO_4_⋅12H_2_O, 130 mM NaHCO_3_, and 5.6 g L^–1^ yeast extract. Prior to operation, D-glucose was added to the cathodic medium. Cathodes were purged with CO_2_, imposed at -0.8 V (vs. Ag/AgCl), and stirred using a magnetic stirrer at 200 rpm.

The cyclic voltammograms (CVs), electrochemical impedance spectroscopy (EIS), and the constant potential [−0.8 V (vs. Ag/AgCl)] were performed using an electrochemical instrument (PMC 1000/DC, AMETEK, Berwyn, PA, United States). The CV scan rate was 20 mV/s in the range from -800 to 600 mV (vs. Ag/AgCl). The EIS experiments (0.01–10^5^ Hz) were carried out at 10 mV amplitude.

### Organic Acid Analysis

Organic acids were analyzed with high-performance liquid chromatography (HPLC) (Agilent 1290; Agilent Technologies, Santa Clara, CA, United States) via an HPX-87H column (300–7.8 mm, Bio-Rad, Hercules, CA, United States), equipped with an ultraviolet spectrophotometric detector (at 215 nm) and a refractive index detector. The mobile phase was 8 mM sulfuric acid and the flow rate was 0.5 mL/min.

### Real-Time PCR Analysis

RNA isolation was prepared using the RNAprep pure cell/bacteria kit (TIANGEN BIOTECH, Beijing, China). RNA concentration was determined using a NanoDrop 2000 spectrophotometer (Thermo Scientific, Waltham, MA, United States), and RNA purity was assessed based on the signals at 230, 260, and 280 nm. The integrity of extracted RNA was determined by agarose gel electrophoresis. RNA was transcribed into cDNA as an RT-PCR template using the PrimeScript RT Master Mix (Perfect Real Time) (Takara, Kusatsu, Shiga, Japan). RT-PCR was performed using SYBR Premix EX Taq (Takara, Kusatsu, Shiga, Japan) and a 7300 Plus Real-Time PCR System (Thermo Scientific, Waltham, MA, United States). *rpoD, ihfB*, and *recA* were used as housekeeping genes (Nitzschke and Bettenbrock, 2018), and gene expression was calculated by relative quantification applying the ΔΔCt method with three replicates. Reaction systems of RT-PCR analysis and gene-specific primers used for quantifying corresponding transcript levels are provided in Supplementary Material. The amplification conditions for RT-PCR analysis were: 95°C for 30 s, followed by 40 cycles each consisting of 95°C for 5 s, 60°C for 34 s, and 60°C for 1 min.

### Proteomic Analysis

#### Protein Extraction, Trypsin Digestion, and Tandem Mass Tag Labeling

The bacterial sample was added to lysis buffer (8 M urea, 1% Protease Inhibitor Cocktail, 3 μM TSA, 50 mM NAM, and 2 mM EDTA) and sonicated on ice three times using a high intensity ultrasonic processor. After centrifugation (12,000 × g, 10 min, 4°C), the supernatant was collected into a new EP tube and the protein concentration was measured according to the manufacturer’s instructions for the BCA kit. For digestion, the protein solution was reduced, alkylated, diluted, and digested by trypsin twice according to a standard protocol reported in previous studies (Li et al., 2018; Zhang et al., 2020). The peptide obtained by trypsin digestion was desalted using a Strata X C18 SPE column (Phenomenex, Torrance, CA, United States) and dried by vacuum. The peptide was reconstituted in 0.5 M triethylammonium bicarbonate (TEAB) and processed with a Tandem Mass Tag (TMT) kit. TMT kits enable multiplex relative quantitation by mass spectrometry (MS). Briefly, one unit of TMT reagent was thawed and reconstituted in acetonitrile. Then TMT label reagent was added to differentially label the sample with six TMT tags (Control group 1–3: 126, 127, and 128 label; MtrCBA group 1–3: 129, 130, and 131 label). After incubation for 2 h at room temperature, the peptide mixtures were pooled, desalted, and dried by vacuum centrifugation.

#### High-Performance Liquid Chromatography Fractionation

The peptides were separated into fractions using high pH reverse-phase HPLC. Thermo Betasil C18 column (5 μm particles, 250 mm, and 10 mm i.d.) was used to perform the HPLC fractionation under a gradient of 8–32% acetonitrile (pH 9.0) within 60 min. Sixty fractions were collected and then combined to 10 fractions. After drying in a vacuum centrifuge, the fractions were dissolved in 0.1% formic acid for liquid chromatography tandem mass spectrometry (LC-MS/MS) analysis.

#### Liquid Chromatography Tandem Mass Spectrometry Analysis

For the collected fractions, peptides were analyzed (three repeats per component) using an EASY-nLC 1000 UPLC system coupled online with Q Exactive™ Plus (Thermo, United States). A home-made reversed-phase analytical column (15 cm length, 75 μm i.d.) was used to separate the peptides. Mobile phases A (2% acetonitrile in 0.1% formic acid) and B (0.1% formic acid in 90% acetonitrile) were employed at a constant flow rate of 500 nL/min by the following method: linear gradient from 9% mobile phase B to 26% over 23 min; linear gradient to 38% mobile phase B in 9 min; 38–80% in 4 min and holding at 80% for 4 min. The mass spectrometry parameters were set as follows: the electrospray voltage was set as 2.0 kV; the m/z scan range was set as 350 to 1,800; and intact peptides were detected at 70,000 resolution in the Orbitrap. Peptides were then selected for MS/MS using NCE setting as 28 and the fragments were detected at 17,500 resolution in the Orbitrap. A data-dependent process was conducted that alternated between one MS scan followed by 20 MS/MS scans with 15.0 s dynamic exclusion. The fixed first mass was 100 m/z.

#### Proteomics Data Analysis and Bioinformatics Methods

The resulting MS/MS data were searched against the *E. coli* K12 database (4,446 sequences) using Maxquant search engine (v.1.5.2.8). The parameters were as follows: cleavage enzyme was trypsin/P; missing cleavages was set to 2; first search peptide tolerance and main search peptide tolerance were 20 and 5 ppm, respectively; the fragment mass tolerance was 0.02 Da; fixed modification was carbamidomethyl, variable modifications were acetylation modification and oxidation on Met. The false discovery rate (FDR) was adjusted to below 1%.

Gene Ontology (GO) proteins were annotated using InterProScan and derived from the UniProt-GOA database,^1^ which were classified according to three categories of GO annotation: biological process, cellular component, and molecular function. The protein pathway annotation was performed using Kyoto Encyclopedia of Genes and Genomes (KEGG) online tool KAAS (KEGG Automatic Annotation Server) based on the KEGG database. Subcellular localization predication software wolfpsort was used to predict subcellular localization. For functional enrichment, enrichment of GO analysis and pathway analysis were researched by employing a two-tailed Fisher’s exact test to test the enrichment of the DEPs against all identified proteins, and the corrected *p*-value < 0.05 is considered significant. Based on DEP functional classification, further hierarchical clustering was conducted, then visualized by a heat map using the “heatmap.2” function from the “gplots” R-package.

## Results

### Effect of the Exogenous Electrons on Metabolite Profiles

The biocathodic BES has been widely applied to convert electrons from electrodes into intracellular reducing power, promoting the production of target metabolites. In the succinate anaerobic fermentation pathway (see Supplementary Material for metabolic pathway), two molecules of NADH are produced from one molecule of glucose into two molecules of intermediate phosphoenolpyruvate (PEP), while four NADH molecules are needed to synthesize two molecules of succinate from two PEP molecules with CO_2_ supplementation. Therefore, insufficient reducing power is the critical limiting factor in succinate yield (Zhu and Tang, 2017; Wu et al., 2019). In our work, succinate production in the BES was investigated and the succinate-producing strain *E. coli* BA102 was used as a host strain. This host strain was constructed to reduce the main byproduct by deleting *pflB* and *ldhA* genes. In addition, the pool of available PEP was increased by disabling the *ptsG* gene encoding an enzyme for the PEP-dependent phosphotransferase system to preserve enhanced succinate production (see Supplementary Material for metabolic pathway) (Cao et al., 2011; Singh et al., 2011; Feng et al., 2020b).

**FIGURE 1:**
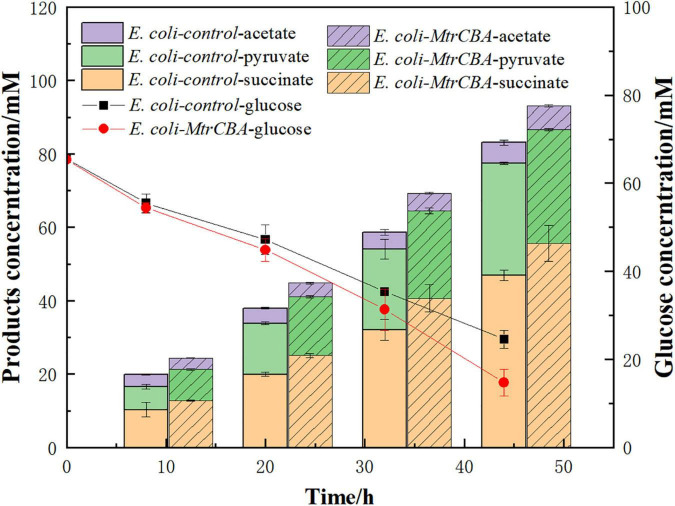
Effects of the exogenous electrons on major metabolite profiles. Bioelectrochemical reactors inoculated with *E. coli-control* or *E. coli-MtrCBA* (OD_600_ = 1) in cathode mediums were imposed at a constant potential [-0.8 V (vs. Ag/AgCl)].

*MtrCAB*-expressing *E. coli* strain *E. coli-Mtr* was constructed by introducing the Mtr pathway from *S. oneidensis* MR-1 and the engineered Mtr pathway allowed electron transfer from the cathode to *E. coli* cells, which realized the use of exogenous electrons (Feng et al., 2020a). To better understand the effect of exogenous electrons on metabolite profiles, *E. coli-MtrCBA* and *E. coli-control* were used in BES with glucose as the carbon source and CO_2_ supplementation. As shown in Figure 1, *E. coli-MtrCBA* had faster glucose consumption and succinate production rates, and the succinate concentration of *E. coli-MtrCBA* was 18% higher than that of *E. coli-control* after 44 h. Pyruvate was accumulated as a result of gene knockouts. Production of the main by-products pyruvate and acetate showed no significant difference between *E. coli-MtrCBA* and *E. coli-control*, while higher levels of succinate/pyruvate and succinate/acetate were shown in *E. coli-MtrCBA* (increased by 17 and 2%, respectively). The results indicated that the intracellular metabolite profiles were stimulated and changed to facilitate the production of reduced end-products (succinate) in the BES driven by electricity (Wu et al., 2019).

### Electrochemical Activity of *Escherichia coli* Cells in the Bioelectrochemical System

**FIGURE 2:**
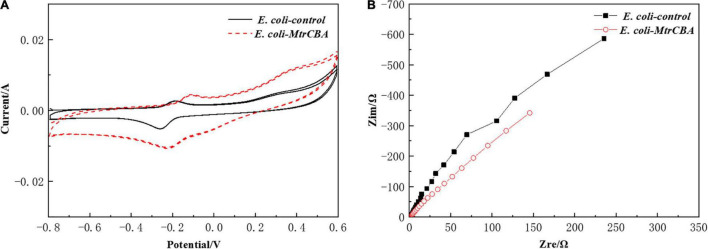
Effects of exogenous electrons on electrochemical activity. Cyclic voltammogram (CV) analysis scanned at 10 mV/s in the range of -800–600 mV **(A)**. Electrochemical impedance spectroscopy scanned at 0.01−10^5^ Hz with a 10 mV amplitude **(B)**.

To analyze the electrochemical performance of the BES, CV curves and nyquist plots of impedance were obtained from CV and EIS test to investigate the redox reaction characteristics between the electrode and the microbial cells. CVs of *E. coli-MtrCBA* and *E. coli-control* are presented in Figure 2A. Two oxidation peaks [ranging from +0.2 to +0.40 V (vs. Ag/AgCl) and −0.20 to 0.0 V (vs. Ag/AgCl)] and one reduction peak between −0.30 and −0.10 V (vs. Ag/AgCl) were observed, consistent with the broad potential of c-type cytochromes ranging from −0.45 to +0.20 V and the mediator molecules excreted by *E. coli* ranging from −0.45 to −0.3 V (Qiao et al., 2008; Ross et al., 2011; Kracke et al., 2015). This demonstrated that the redox peaks were caused by c-type cytochromes and endogenous redox species under the effect of exogenous electrons (Feng et al., 2020a). The higher peak area obtained from *E. coli-MtrCBA* compared with the control indicated that stronger redox reactions occurred in the BES inoculated with *E. coli-MtrCBA.* Moreover, the oxidation and reduction peaks of *E. coli-MtrCBA* shifted positively compared with the control group. Since proton transfer coupled with electron transfer is involved in the redox reaction, the CV result is affected by solution pH value. A previous report showed that the midpoint potential of the redox peak shifts positively with decreased pH (Qiao et al., 2008). More succinic acid produced by *E. coli-MtrCBA* led to a lower pH, resulting in a slightly positive shift in the redox peaks of *E. coli-MtrCBA.* In addition, the electron transfer efficiency at the cell-electrode interfaces was analyzed by EIS. EIS analysis reflected an improved electron transfer rate of *E. coli-MtrCBA* (Figure 2B) (Liu et al., 2012). These results confirmed that the engineered *E. coli* exhibited much better electrochemical activity and more effective biocatalytic behavior in BES compared to control.

### Real-Time PCR Analysis of Specific Gene Expression Changes

**FIGURE 3:**
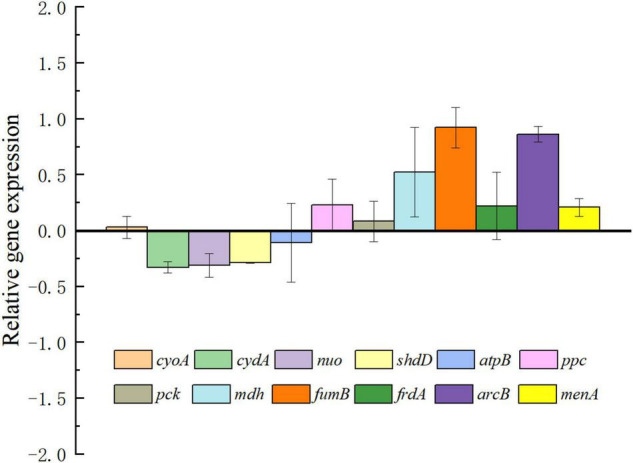
Results of RT-PCR analysis. The transcription pattern was normalized to the reference genes for the expression of *E. coli-control*. Genes encoding cytochrome bo3 (*cyoA*), cytochrome bd (*cydA*), NADH: ubiquinone oxidoreductase (*nuo*) and succinate dehydrogenase (*sdhD*), ATP synthase (*atpB*), phosphoenolpyruvate carboxylase (*ppc*), phosphoenolpyruvate carboxykinase (*pck*), malate dehydrogenase (*mdh*), fumarate hydratase (*fumB*), fumarate reductase (*frdA*), sensor histidine kinase (*arcB*), and 1,4-dihydroxy-2-naphthoate octaprenyltransferase (*menA*) were analyzed. The unchanged relative gene expression level was calculated as 0. The Y-axis was formatted in a logarithmic scale with base 10.

To analyze the effect of exogenous electrons transported into cells by intracellular metabolic pathways, the expression changes of important genes related to terminal oxidases, respiratory chain, and the tricarboxylic acid (TCA) cycle were investigated via RT-PCR. The relative expression levels for selected genes are shown in Figure 3. As expected, genes connected to the TCA cycle including *fum* and *mdh* were upregulated in *E. coli-MtrCBA.* This is consistent with more succinate being produced by *E. coli-MtrCBA* through accepting cathodic electrons, hinting at a more active succinate synthesis pathway. There was an obvious change in expression level of intracellular redox regulation system protein gene *arcB*. The Arc two-component system comprising ArcA and ArcB is the key element in the global transcriptional regulatory network affecting the expression of numerous genes associated with substrate metabolism and energy metabolism to adapt to various conditions (van Beilen and Hellingwerf, 2016; Teran-Melo et al., 2018). Van Beilen and Hellingwerf reported that reduced quinone species could activate ArcB as a redox-sensitive, membrane-embedded kinase (van Beilen and Hellingwerf, 2016). These results suggested that exogenous electrons might affect a wide range of intracellular gene expression and expressed proteins and then biological reactions and pathways.

### Comprehensive Proteomics Analysis of Electron Utilization in *Escherichia coli* Cells

The mechanism of electron transfer and utilization is complex and not fully understood. To reveal key intracellular proteins or pathways responding to exogenous electrons and further comprehensively analyze the effect of electron transfer and utilization, systematic proteomic analysis research was conducted.

#### Tandem Mass Tag Analysis of Differentially Expressed Proteins

**FIGURE 4:**
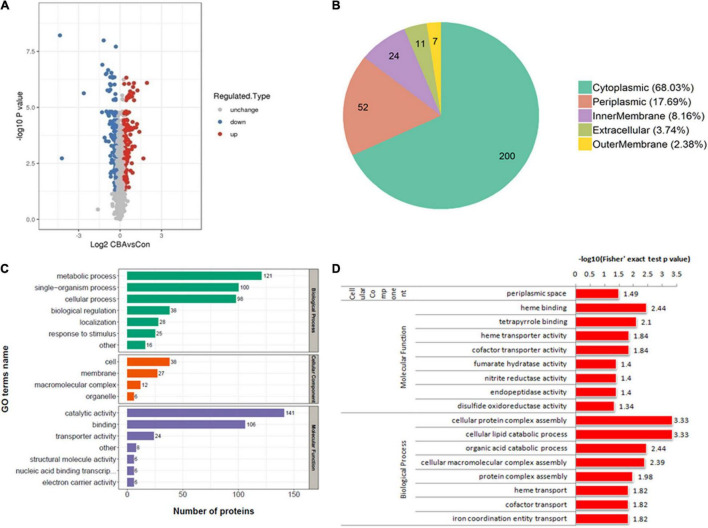
Results of proteomic analysis. The volcano plot shows the upregulated (red) or downregulated (blue) proteins between *E. coli-MtrCBA/E. coli-control* **(A)**. Subcellular localization map of the differentially expressed proteins **(B)**. The differentially expressed proteins classified according to Gene Ontology (GO) annotation **(C)**. GO enrichment analysis of the differentially expressed proteins **(D)**. –Log 10 *P* value represents the negative logarithm of *p* value calculated by Student’s *t*-test to base 10. Log 2 CBAvsCon represents the logarithm of the proteins’ difference multiple to the base 2. –Log10 (Fisher’s exact test *p* value) represents the negative logarithm of *p* value calculated by the Fisher’s exact test to base 10.

In total, 2,268 proteins were detected by TMT quantitative proteomics. Of these, 2,110 proteins were identified as quantifiable DEPs. Based on a change threshold of 1.2-fold, the results of protein expression levels are shown in the volcano plot in Figure 4A. Compared with the control, exogenous electrons from the cathode led to up-regulation of 152 proteins and down-regulation of 142 proteins in *E. coli-MtrCBA*.

#### Gene Ontology Annotation and Enrichment Analysis

In order to acquire protein functional information, the DEPs were annotated and classified by GO annotation according to three categories (biological process, cellular component, and molecular function) and subcellular location (Figures 4B,C). Among them, the DEPs were respectively located in the cytoplasm (200 proteins, accounting for 68%), the periplasm (52 proteins, accounting for 18%), the innermembrane (24 proteins, accounting for 8%), the extracellular matrix (11 proteins, accounting for 4%), and the outermembrane (seven proteins, accounting for 2%).

Based on GO annotations and classification into three categories, GO enrichment analysis was performed using a two-tailed Fisher’s exact test to identify significantly enriched functional groups (*p*-value < 0.05) of the DEPs in the case of exogenous electron transfer into *E. coli* cells through the Mtr pathway (Figure 4D). In terms of cellular components, the DEPs were highly significantly enriched in the perplasmic space. MtrCAB complexes that span the outer membrane allow electron transfer from the decaheme cytochrome MtrC located on the extracellular face of the outer membrane to the periplasmic decaheme cytochrome MtrA, so proteins in the periplasmic space are directly affected by electrons (Jensen et al., 2010). DEPs were highly significantly categorized into eight biological processes related to the metabolism of protein, lipid, and organic acid, including “cellular protein complex assembly,” “cellular lipid catabolic process,” “cellular macromolecular complex assembly,” “protein complex assembly,” and “organic acid catabolic process,” and associated with GO terms about electrons, including “heme transport,” “cofactor transport,” and “iron coordination entity transport.” Among molecular function-associated GO terms, the main enriched molecular functions of the DEPs were related to binding and transport, including “heme binding,” “tetrapyrrole binding,” “heme transporter activity,” and “cofactor transporter activity.” MtrC and MtrA, the important parts of MtrCAB complexes, are decaheme cytochrome c, and their maturations are required to load heme (Jensen et al., 2010). Cofactors, such as NADH, MKH_2_, and FADH, are intracellular electron acceptors, which are used to store reducing power. In addition, “nitrite reductase activity” and “disulfide oxidoreductase activity,” which functionally catalyze the reactions commonly involved in electrons, were significantly enriched.

#### Enrichment-Based Clustering Analysis

**FIGURE 5:**
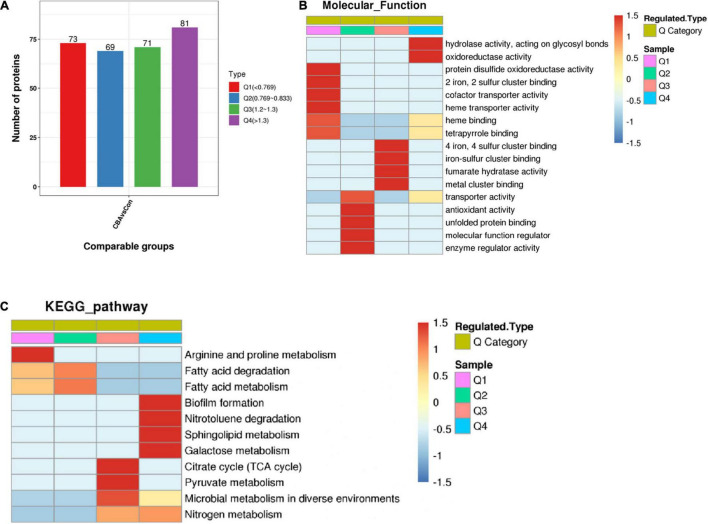
Enrichment-based clustering analysis. The differentially expressed proteins were divided into four parts: Q1 (<0.769), Q2 (0.769–0.833), Q3 (1.2–1.3), and Q4 (>1.3) according to differential expression multiple **(A)**. Hierarchical clustering based on functional classification of differentially expressed proteins (Molecular Function) **(B)** and KEGG Pathway based on *Escherichia coli* K-12 MG1655 **(C)**. Color from blue to red represents the degree of enrichment from weak to strong, respectively.

On the basis of enrichment analysis, further cluster analysis was carried out to find correlations or new insights into protein functions and pathways. According to differential expression multiple, the DEPs were divided into four parts: Q1, Q2, Q3, and Q4 (Figure 5A) and then clustered by hierarchical clustering. The results were visualized by heat maps.

A cluster analysis heat map based on molecular function enrichment is shown in Figure 5B. “Hydrolase activity” and “oxidoreductase activity” (Q4 > 1.3) were significantly enhanced by exogenous electrons transferred into *E. coli* cells through the Mtr pathway, functionally catalyzing the reactions commonly accompanied with electrons or protons. DEPs of molecular function associated with “4 iron, 4 sulfur([4Fe-4S]) cluster binding,” “iron-sulfur (Fe-S) cluster binding,” and “metal cluster binding” (1.2 ≤ Q3 ≤ 1.3) were up-regulated, while those associated with “2 iron, 2 sulfur ([2Fe-2S]) cluster binding” (Q4 < 0.769) were down-regulated (Supplementary Material). Fe-S clusters are versatile and essential biological cofactors biosynthesized by the Isc and Suf pathways in *E. coli* (Mettert and Kiley, 2015). Reducing power such as in the presence of the reducing agent DTT is absolutely required for mature [4Fe-4S] proteins, due to the requirement of electrons to generate [4Fe-4S] from [2Fe-2S] clusters (Chandramouli et al., 2007; Beilschmidt et al., 2017). Electrons were transferred from the cathode into *E. coli* cells through the Mtr pathway, resulting in a reductive intracellular environment and providing electrons for [4Fe-4S] generation. This further promoted [4Fe-4S] cluster binding and [4Fe-4S] protein maturation.

Fe-S cluster-containing proteins are partly reductases and dehydrogenases, in which Fe-S clusters are the relay station or conduit participating in electron transfer. Fe-S clusters also serve as prosthetic groups acting as binding sites or participating in enzyme catalysis. In addition, the up-regulation of “fumarate hydratase activity” suggested that it promoted formation of fumarate, the precursor of succinate. Fumarate hydratase is also a [4Fe-4S] cluster-containing protein, in which the [4Fe-4S] cluster is used for substrate binding and readily oxidized to an inactive [3Fe-4S] cluster (Flint et al., 1992). A reductive intracellular environment caused by exogenous electrons could promote binding and stable activity of [4Fe-4S] clusters. Unexpectedly, the DEPs were significantly enriched in terms of negative regulation for “heme transporter activity,” “heme binding,” and “cofactor transporter activity.” Further analysis of the specific DEPs showed that CcmC and CcmF were the main DEPs in three terms related to the synthesis and maturation of the MtrCAB complexes. MtrCAB complexes spanning the outer membrane contain two cytochrome c MtrA and MtrC, whose maturation is required for cytochrome c maturation proteins CcmABCDEFGH (Jensen et al., 2010; Feng et al., 2020a). The polypeptides of MtrCAB and CcmABCDEFGH are secreted into the periplasm by the Sec secretion system, and then heme is translocated into the periplasm and loaded to form mature MtrC and MtrA by Ccm machinery. During synthesis and maturation of the MtrCAB complexes, there are multiple regulation points: excessive production or secretion of Mtr can inhibit and decrease Ccm, and inadequate heme production can lead to degradation of apocytochrome c or cytochrome c (Goldbeck et al., 2013). These might be the reasons why heme binding and transporter activity were down-regulated.

A cluster analysis heat map based on KEGG pathway enrichment is shown in Figure 5C. Among these metabolic pathways, DEPs are mainly connected with biofilm and cell membrane, such as the up-regulation of biofilm formation and sphingolipid metabolism (Q > 1.3), and down-regulation of fatty acid metabolism and fatty acid degradation (0.769 ≤ Q ≤ 0.833). These findings indicated that biofilm formation and cell membrane composition were significantly affected. The TCA cycle, pyruvate metabolism, and nitrogen metabolism pathway were promoted, which allowed the cells to produce more succinate (Feng et al., 2020a).

### The Channel Protein Promoted Redox Reactions Between the Electrode and *Escherichia coli* Cells

The results of electrochemical analysis confirmed that *E. coli-MtrCBA* exhibited much better bioelectrocatalytic activity and catalyzed stronger redox reactions compared with the control in the BES. The contributing factor was endogenous molecules under electrochemical stress (Feng et al., 2020a). Moreover, proteomic analysis indicated that the cell membranes were affected during the process of electron utilization in the BES. Combined with the data of subcellular location, seven of the significant DEPs were located in the outer membrane. Excluding uncharacterized proteins, the up-regulated outer membrane protein OmpF (*E.coli-MtrCBA/E.coli-control* Ratio = 1.653) was further identified. OmpF is a highly abundant primary porin in *E. coli*, which enables passive diffusion of hydrophilic molecules and influences outer membrane permeability (Duval et al., 2017).

**FIGURE 6:**
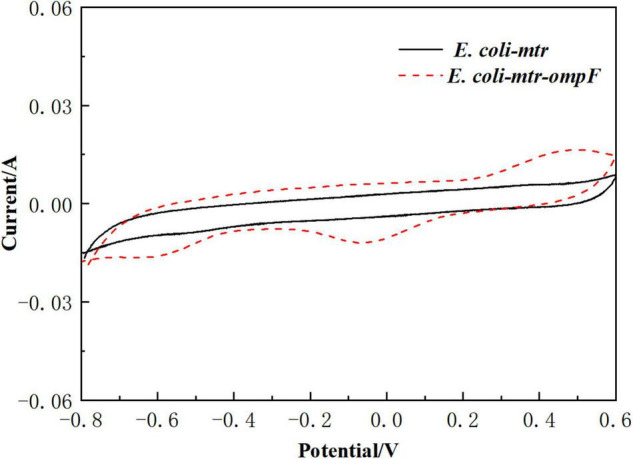
CV analysis of *E. coli-mtr* and *E. coli-mtr-ompF* scanned from -800 to 600 mV.

To determine that the channel protein OmpF assisted with the redox reactions between the electrode and *E. coli* cells, *ompF* overexpressed strain *E. coli-mtr-ompF* and control *E. coli-mtr* were constructed and inserted into the BES to perform a CV test. As shown in Figure 6, significant redox peaks were observed from the BES inoculated with *E. coli-mtr-ompF*, indicating that OmpF might reduce transmission resistance of the cell membrane and improve outer membrane permeability to enhance the redox reactions. This was one of the reasons for the better bioelectrocatalytic activity exhibited by the engineered *E. coli-MtrCBA.*

## Discussion

The process by which microorganisms utilize electrons from a cathode to produce high-value fuels and chemicals is the basis of the biocathodic BES. However, major knowledge gaps on molecular mechanisms of electron transfer and the effect on microbial cells have limited further engineering of microorganisms and practical applications of biocathodic BES. In order to utilize the electrons of cathodes, *E. coli* engineered with the Mtr pathway was constructed by expressing *mtrCAB* from *S. oneidensis* MR-1. The engineered *E. coli* exhibited much better electrochemical activity and more effective biocatalytic behavior than control in BES based on CV and EIS analyses. The Mtr pathway in *S. oneidensis* MR-1 is a typical bidirectional electron transfer pathway, in which electrons flow from cells into anodes or from cathodes into cells (Ross et al., 2011; Feng et al., 2020a). Moreover, the Mtr pathway has been successfully constructed in *E. coli* (Jensen et al., 2010; Goldbeck et al., 2013; Wu et al., 2019). The electrochemical performance of *E. coli-MtrCBA* indicated that our engineered *E. coli* cells could transfer electrons from the electrodes to cells to utilize exogenous electrons. Compared with *E. coli-control*, the electroactive *E. coli-MtrCBA* could boost succinate production by 18% and displayed higher levels of succinate/pyruvate and succinate/acetate (enhanced by 17 and 2% respectively) in BES driven by electricity. The results suggested that *E. coli-MtrCBA* accepted electrons from the cathode through the Mtr pathway and utilized the electrons to obtain excess reducing power, which led to an imbalance of the intracellular redox state and thus metabolite profiles were stimulated and changed to facilitate the production of reduced products (Wu et al., 2019).

Subsequently, systematic proteomic analysis research and RT-PCR were performed to reveal key intracellular proteins or pathways in response to exogenous electrons and comprehensively analyze the effect of electron utilization on *E. coli* cells. Compared with the control, 152 up-regulated and 142 down-regulated proteins were identified in *E. coli-MtrCBA*, respectively. Bioinformatics analysis showed that the proteins of molecular function associated with hydrolase activity, oxidoreductase activity, [4Fe-4S] cluster binding, Fe-S cluster binding, metal cluster binding, and fumarate hydratase activity were positively affected by exogenous electrons. In addition to the proteins that functionally catalyze reactions involving electrons or protons, the Fe-S cluster was worth further study. Fe-S cluster-containing proteins are partly electron-accepting terminal oxidoreductases and dehydrogenases participating in multiple cellular processes, and Fe-S clusters are the relay station used to exchange electrons within the proteins (de Choudens and Barras, 2017; Feng et al., 2020a). Fe-S clusters can also function as prosthetic groups acting as the binding sites or participating in enzyme catalysis. Due to the requirement of electrons to generate [4Fe-4S] from [2Fe-2S] clusters, sufficient reducing power was absolutely required to generate mature [4Fe-4S] proteins and also stable activity of [4Fe-4S] clusters (Chandramouli et al., 2007; Beilschmidt et al., 2017).

Up-regulated proteins were mainly involved in the KEGG pathways of TCA, pyruvate metabolism, and nitrogen metabolism pathway, showing that the metabolic balance of microbial cells shifted toward reduced end-products due to electron utilization. Moreover, the DEPs enriched in sphingolipid metabolism and fatty acid metabolism indicated that cell membranes were affected during the process of electron utilization in BES. The *ompF* overexpressed strain was used to investigate the function of the channel protein OmpF located in the outer membrane. CV analysis showed that the up-regulated expression of *ompF* improved redox reactions between the electrode and the cells.

In conclusion, engineered *E. coli* transferred electrons from the cathode into the cells by the Mtr pathway and simultaneously up-regulated the channel protein to enhance redox reactions between the electrode and the cells to further improve electron transfer capacity. The electrons were transferred into *E. coli* cells, resulting in a reductive intracellular environment, which provided a basis to improve the maturation or stability of some proteins associated with “[4Fe-4S] cluster binding,” “Fe-S cluster binding,” and “metal cluster binding.” Sufficient reducing power provided the cofactor and electron donor for the oxidoreductases and dehydrogenases. The DEPs induced by exogenous electrons were mapped to the KEGG pathway database, which showed that the electrons disturbed metabolism in multiple ways by biofilm formation, sphingolipid metabolism, fatty acid metabolism, TCA, pyruvate metabolism, and nitrogen metabolism pathway.

## Conclusion

In this work, *E. coli* engineered to contain the Mtr pathway exhibited better electrochemical activity and effective biocatalytic behavior. Based on electrochemical and metabolite profile analyses, the succinate yield increased by 18% showing the effect of exogenous electrons on *E. coli* cells. Subsequently, proteomics and RT-PCR were performed to investigate DEPs in *E. coli* in response to exogenous electrons. Based on bioinformatic analysis of the DEPs, combined with biochemical methods, potential proteins and metabolic pathways that might function in electron transfer and utilization of *E. coli* cells were identified. In future studies, more crucial gene targets for electron transfer and utilization need to be identified. Additionally, multi-level and large-scale connections should be explored. The detailed molecular mechanisms need much deeper research, as an in-depth understanding of molecular mechanisms is the basis for engineering microorganisms to improve electron utilization efficiency. This work revealed a global proteomic alteration of *E. coli* cells in response to exogenous electrons, which will aid in the understanding of molecular mechanisms on electron transfer and utilization, and provide a theoretical basis for improving electron transfer and utilization efficiency.

## Data Availability Statement

The datasets presented in this study can be found in online repositories. The names of the repository and accession number can be found below: http://proteomecentral.proteomexchange.org/cgi/GetDataset?ID=PXD030650.

## Author Contributions

JiaoF: data curation, validation, investigation, writing–original draft, review, and editing, and funding acquisition. JiaF: data curation, formal analysis, and writing–review and editing. CL: data curation and writing–review and editing. SX: resources, project administration, funding acquisition, and writing–review and editing. XW and KC: resources and writing–review and editing. All authors contributed to the article and approved the submitted version.

## Conflict of Interest

The authors declare that the research was conducted in the absence of any commercial or financial relationships that could be construed as a potential conflict of interest.

## Publisher’s Note

All claims expressed in this article are solely those of the authors and do not necessarily represent those of their affiliated organizations, or those of the publisher, the editors and the reviewers. Any product that may be evaluated in this article, or claim that may be made by its manufacturer, is not guaranteed or endorsed by the publisher.

## Footnotes

1
http://www.ebi.ac.uk/GOA/
