# Supplementary material for: Proteomics Reveal the Effect of Exogenous Electrons on Electroactive Escherichia coli
Source: Front Microbiol. 2022 Apr 6;13:815366. doi: 10.3389/fmicb.2022.815366 (PMC9019752; doi:10.3389/fmicb.2022.815366)
Supplement: Supplementary file 1 [file Image_1.pdf]

Supplementary Material

Proteomics reveal the effect of exogenous electrons on electroactive *Escherichia coli*

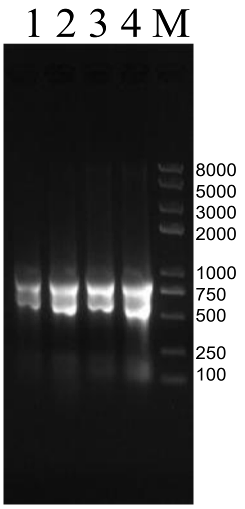

Figure S1 Agarose gel electrophoresis of RNA. 1and 3: RNA of *E. coli-control*; 2and 4: RNA of *E. coli-MtrCBA*; M: Trans2K Plus DNA Marker
