# Supplementary material for: Proteomics Reveal the Effect of Exogenous Electrons on Electroactive Escherichia coli
Source: Front Microbiol. 2022 Apr 6;13:815366. doi: 10.3389/fmicb.2022.815366 (PMC9019752; doi:10.3389/fmicb.2022.815366)
Supplement: Supplementary file 2 [file Image_2.pdf]

Supplementary Material

Proteomics reveal the effect of exogenous electrons on electroactive *Escherichia coli*

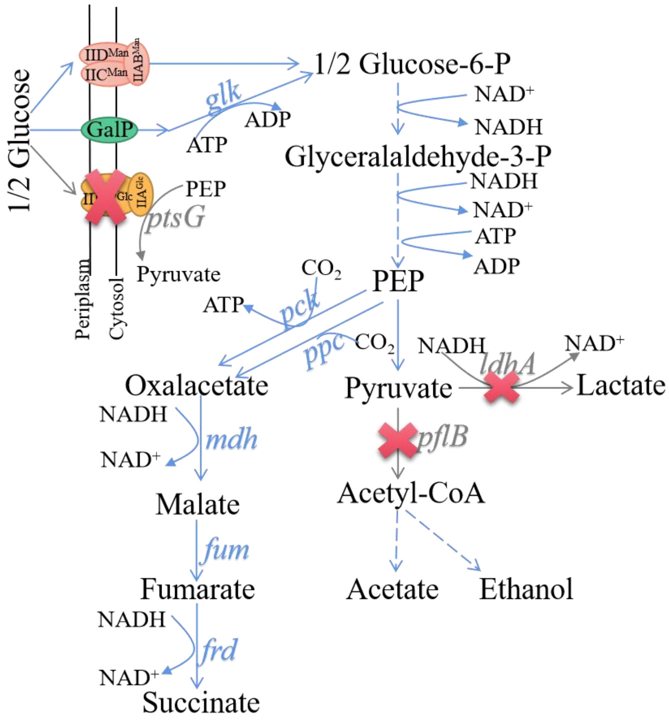

Figure S2 Central metabolic pathway of *E. coli* for succinate production in this work. PEP: phosphoenolpyruvate. Dashed lines represent multi-step reactions. Gene deletions are indicated in gray and crossed out.
