# Supplementary material for: Proteomics Reveal the Effect of Exogenous Electrons on Electroactive Escherichia coli
Source: Front Microbiol. 2022 Apr 6;13:815366. doi: 10.3389/fmicb.2022.815366 (PMC9019752; doi:10.3389/fmicb.2022.815366)
Supplement: Supplementary file 3 [file Table_1.DOCX]

**Supplementary Material**

**Proteomics reveal the effect of exogenous electrons on electroactive *Escherichia coli***

Table S1 Primers in this work

| Primers | Oligonucleotides |
| --- | --- |
| ompF-1 | CCGGAATTCATGATGAAGCGCAATATTC (*Eco* RI) |
| ompF-2 | CGAGCTCTTAGAACTGGTAAACGATAC (*Sac* I) |
| RT-PCR |  |
| frdA-1 | GGGTGAATGTTCCTCTGTTG |
| frdA-2 | CACGCTCTGTCGCTTGTT |
| cydA-1 | CTTACAGTTTGCCTTGACC |
| cydA-2 | CCCAGAGCGAAGTTGATA |
| mdh-1 | TTATTGGCGGTCACTCTG |
| mdh-2 | GTTCTGGATGCGTTTGG |
| sdhD-1 | ATCGGTTTCTTCGCCTCTG |
| sdhD-2 | CGGTCAACACCTGCCACAT |
| cyoA-1 | AGTGACCTCCAACTCCGTGAT |
| cyoA-2 | GGTGTTCGGCGACTGCTT |
| nuo-1 | CGGTATCCCGATGACGC |
| nuo-2 | CCACCAGCCACCACAAG |
| nap-1 | CCGTAACTGTCACAACTTCG |
| nap-2 | GTTTGCCCATCTTTCACC |
| arcB-1 | CGTTCTTGCCATTGTG |
| arcB-2 | ACCACCGATAGAAAGTAGA |
| ihfB-1 | GCCAAGACGGTTGAAGATGC |
| ifhB-2 | GAGAAACTGCCGAAACCGC |
| recA-1 | CGCTTGGGGCAGGTGGTCT |
| recA-2 | TGCAGCGTCAGCGTGGTTTT |
| rpoD-1 | TCGCCAAAGAGCCAATC |
| rpoD-2 | GCGTGTAGTCGGTGTTCATA |
| fumB-1 | GACGGAAGGGAACGAAC |
| fumB-2 | GTGGCAGACGGATAACG |
| ppc-1 | GCGTAACCGAACAGGG |
| ppc-2 | GCGGAAGTAAGGCACAA |
| pck-1 | GGGCAGACAAAGGCAAAG |
| pck-2 | AACGGACGGAAAGACGAG |
| atpB-1 | GCAGGATTACATAGGACACC |
| atpB-2 | CAGCACCACCGAGAAGA |
