# Supplementary material for: Proteomics Reveal the Effect of Exogenous Electrons on Electroactive Escherichia coli
Source: Front Microbiol. 2022 Apr 6;13:815366. doi: 10.3389/fmicb.2022.815366 (PMC9019752; doi:10.3389/fmicb.2022.815366)
Supplement: Supplementary file 4 [file Table_2.DOCX]

**Supplementary Material**

**Proteomics reveal the effect of exogenous electrons on electroactive *Escherichia coli***

Table S2 Purity detection of RNA

| Strains | Concentration（μg/mL） | A260 | A280 | A260/A280 | A260/A230 |
| --- | --- | --- | --- | --- | --- |
| *E. coli-control* | 577.2 | 14.43 | 6.80 | 2.12 | 2.30 |
| *E. coli-MtrCBA* | 1504.2 | 37.61 | 17.48 | 2.15 | 2.29 |
