# Supplementary material for: Proteomics Reveal the Effect of Exogenous Electrons on Electroactive Escherichia coli
Source: Front Microbiol. 2022 Apr 6;13:815366. doi: 10.3389/fmicb.2022.815366 (PMC9019752; doi:10.3389/fmicb.2022.815366)
Supplement: Supplementary file 5 [file Table_3.DOCX]

**Supplementary Material**

**Proteomics reveal the effect of exogenous electrons on electroactive *Escherichia coli***

Table S3 Reaction system of real-time PCR

| Composition | Volume |
| --- | --- |
| SYBR *Premix Ex Taq*(2×) (Tli RNaseH Plus) | 10 μL |
| ROX Reference Dye(50×) | 0.4 μL |
| Primer1 | 0.4 μL |
| Primer2 | 0.4 μL |
| DNA template | 2 μL |
| ddH_2_O | 6.8 μL |
