# Supplementary material for: Proteomics Reveal the Effect of Exogenous Electrons on Electroactive Escherichia coli
Source: Front Microbiol. 2022 Apr 6;13:815366. doi: 10.3389/fmicb.2022.815366 (PMC9019752; doi:10.3389/fmicb.2022.815366)
Supplement: Supplementary file 6 [file Table_4.docx]

**Supplementary Material**

**Proteomics reveal the effect of exogenous electrons on electroactive *Escherichia coli***

Table S4 Proteomic analysis of 7 differentially expressed proteins located in the outermembrane

| Protein Accession | Protein | CBA/Con Ratio | P value | Gene |
| --- | --- | --- | --- | --- |
| P0AFS9 | Murein DD-endopeptidase MepM | 0.593 | 5.55E-05 | *mepM* |
| P0AGK8 | HTH-type transcriptional regulator IscR | 0.687 | 2.84E-03 | *iscR* |
| P69856 | Probable N-acetylneuraminic acid outer membrane channel protein NanC | 0.162 | 2.34E-06 | *nanC* |
| P02931 | Outer membrane protein F | 1.653 | 3.61E-4 | *ompF* |
| P03023 | Lactose operon repressor | 0.722 | 1.81E-05 | *lacI* |
| P76116 | Uncharacterized protein YncE | 1.209 | 3.75E-05 | *yncE* |
| P31828 | Probable zinc protease PqqL | 0.546 | 1.90E-05 | *pqqL* |
