# Supplementary material for: Proteomics Reveal the Effect of Exogenous Electrons on Electroactive Escherichia coli
Source: Front Microbiol. 2022 Apr 6;13:815366. doi: 10.3389/fmicb.2022.815366 (PMC9019752; doi:10.3389/fmicb.2022.815366)
Supplement: Supplementary file 7 [file Table_5.DOCX]

**Supplementary Material**

**Proteomics reveal the effect of exogenous electrons on electroactive *Escherichia coli***

Table S5 Proteomic analysis of the differentially expressed proteins associated with iron-sulfur cluster binding

| GO Terms ID | Protein Accession | Protein Description | Gene | CBA/Con Ratio | P value |
| --- | --- | --- | --- | --- | --- |
| GO Terms Description: iron-sulfur cluster binding | | | | | |
| GO:0051536 | P76440 | *NAD-dependent dihydropyrimidine dehydrogenase subunit* | *preT* | 1.280 | 8.25E-05 |
| GO:0051536 | P33937 | *Periplasmic nitrate reductase* | *napA* | 1.286 | 1.23E-04 |
| GO:0051536 | P75824 | *NADH oxidoreductase* | *hcr* | 1.248 | 5.74e-05 |
| GO:0051536 | Q46861 | *UPF0313 protein YgiQ* | *ygiQ* | 1.227 | 4.57E-04 |
| GO:0051536 | P0A996 | *Anaerobic glycerol-3-phosphate dehydrogenase subunit C* | *glpC* | 1.250 | 6.42E-05 |
| GO:0051536 | P0AEI4 | *Ribosomal protein S12 methylthiotransferase* | *rimO* | 1.206 | 4.23E-05 |
| GO:0051536 | P0AEI1 | *tRNA-2-methylthio-N(6)-dimethylallyladenosine synthase* | *miaB* | 1.217 | 1.78E-04 |
| GO Terms Description:4 iron, 4 sulfur cluster binding | | | | | |
| GO:0051539 | P33937 | *Periplasmic nitrate reductase* | *napA* | 1.286 | 1.23E-04 |
| GO:0051539 | Q46861 | *UPF0313 protein YgiQ* | *ygiQ* | 1.227 | 4.57E-04 |
| GO:0051539 | P0AEI4 | *Ribosomal protein S12 methylthiotransferase* | *rimO* | 1.206 | 4.23E-05 |
| GO:0051539 | P0AEI1 | *tRNA-2-methylthio-N(6)-dimethylallyladenosine synthase* | *miaB* | 1.217 | 1.78E-04 |
| GO Terms Description: 2 iron, 2 sulfur cluster binding | | | | | |
| GO:0051537 | P77165 | *Aldehyde oxidoreductase iron-sulfur-binding subunit PaoA* | *paoA* | 0.571 | 2.37E-05 |
| GO:0051537 | P77243 | *2-methylcitrate dehydratase* | *prpD* | 0.505 | 1.51E-05 |
